# Aggregation of Human Trophoblast Cells into Three-Dimensional Culture System Enhances Anti-Inflammatory Characteristics through Cytoskeleton Regulation

**DOI:** 10.3390/ijms19082322

**Published:** 2018-08-08

**Authors:** Kotomi Seno, Yasuhisa Munakata, Michiya Sano, Ryouka Kawahara-Miki, Hironori Takahashi, Akihide Ohkuchi, Hisataka Iwata, Takehito Kuwayama, Koumei Shirasuna

**Affiliations:** 1 Laboratory of Animal Reproduction, Department of Animal Science, Tokyo University of Agriculture, Atsugi, Kanagawa 243-0034, Japan; s11079@yahoo.co.jp (K.S.); 43617002@nodai.ac.jp (Y.M.); 43517012@nodai.ac.jp (M.S.); h1iwata@nodai.ac.jp (H.I.); takehito@nodai.ac.jp (T.K.); 2 NODAI Genome Research Center, Tokyo University of Agriculture, Setagaya, Tokyo 154-0017, Japan; r3miki@nodai.ac.jp; 3 Department of Obstetrics and Gynecology, Jichi Medical University, Shimotsuke, Tochigi 329-0498, Japan; hironori@jichi.ac.jp (H.T.); okuchi@jichi.ac.jp (A.O.)

**Keywords:** inflammation, interleukin-6, regnase-1, trophoblast cells

## Abstract

Background: Three-dimensional (3D) culture changes cell characteristics and function, suggesting that 3D culture provides a more physiologically relevant environment for cells compared with 2D culture. We investigated the differences in cell functions depending on the culture model in human trophoblast cells (Sw.71). Methods: Sw.71 cells were incubated in 2D monolayers or simple 3D spheroids. After incubation, cells were corrected to assess RNA-seq transcriptome or protein expression, and culture medium were corrected to detect cytokines. To clarify the role of actin cytoskeleton, spheroid Sw.71 cells were treated mycalolide B (inhibitor of actin polymerization) in a 3D culture. Results: RNA-seq transcriptome analysis, results revealed that 3D-cultured cells had a different transcriptional profile compared with 2D-cultured cells, especially regarding inflammation-related molecules. Although interleukin-6 (IL-6) mRNA level was higher in 3D-culured cells, its secretion levels were higher in 2D-cultured cells. In addition, the levels of mRNA and protein expression of regnase-1, regulatory RNase of inflammatory cytokine, significantly increased in 3D culture, suggesting post-translational modification of *IL-6* mRNA via regnase-1. Treatment with mycalolide B reduced cell-to-cell contact to build 3D formation and increased expression of actin cytoskeleton, resulting in increased IL-6 secretin. Conclusion: Cell dimensionality plays an essential role in governing the spatiotemporal cellular outcomes, including inflammatory cytokine production and its negative regulation associated with regnase-1.

## 1. Introduction

The placenta is a vital organ during pregnancy that mediates the exchange of gases, nutrients, and waste products between the mother and fetus. It produces various cytokines to regulate placental function, thereby acting as a physiological and immunological barrier between maternal and fetal compartments. The human hemochorial placenta is primarily composed of specialized cells known as trophoblasts.

Studies have been conducted using different methods to elucidate human placental function; however, it is difficult to accurately reflect biological conditions. Murine models can be highly informative, but it is difficult to translate data from animals to humans considering that placenta is species-specific mammalian organ with unique anatomical and physiological properties [1,2]. Experimental models such as tissue culture or ex vivo perfusion of the human placenta have been established. These models have certain drawbacks, such as restricted experimental time of approximately 6–8 h [3,4]. Two-dimensional (2D) in vitro monolayer cell culture using plastic petri dishes is an efficient and a beneficial experimental model. However, 2D-cultured cells lack the complex organization and environment of the placental tissue, including the close interaction of different cell types [5]. Therefore, there is an urgent need for a predictive organotypic in vitro model of the human placenta.

The culturing of many cell types in three-dimensional (3D) has provided an excellent model to mimic the morphological and functional features of cells and tissues in vivo [6]. 3D models can better mimic cell-to-cell and cell-to-matrix interactions [7]. McConkey et al. [6] reported that a 3D-based culture model using human JEG-3 trophoblast cells with endothelial cells exhibited morphological and secretory activities strikingly similar to those of that using primary human syncytiotrophoblasts. In addition, Muoth et al. [5] reported that human chorionic gonadotropin secretion levels were significantly higher in the 3D (placental fibroblasts surrounded by a trophoblast cells) than those in a 2D culture. These findings collectively suggest that 3D culture models have the potential to close the gap between the observed 2D cell culture results and animal studies to enhance the understanding of placental physiology.

In the present study, we developed a simple 3D culture model using human first-trimester trophoblast cells (Sw.71, trophoblast cell line) via use of cell-repellent, surface-treated-cell culture plates. Sw.71 cells abundantly produce cytokines and control regulatory T cell differentiation [8,9]. However, little is known about 3D culture of Sw.71 cells, unlike other trophoblast cell lines (JEG-3, HTR8, or BeWo cells) [2,6]. In the present study, on the basis of our RNA-seq transcriptome analysis, 3D-cultured spheroid Sw.71 cells had a distinct transcriptional profile compared with 2D-cultured Sw.71 cells, especially regarding inflammation-related molecules. Interestingly, because cytokine gene expression and secretion differed between 2D- and 3D-cultured cells, there is a possibility that post-translational modification is active in 3D-spheroid cells. In addition, the cytoskeleton may regulate spheroid formation and inflammatory cytokine production in 3D-cultured Sw.71 cells.

## 2. Results

### 2.1. Aggregation of Human Trophoblast Cells in 3D Cultures

**Figure 1:**
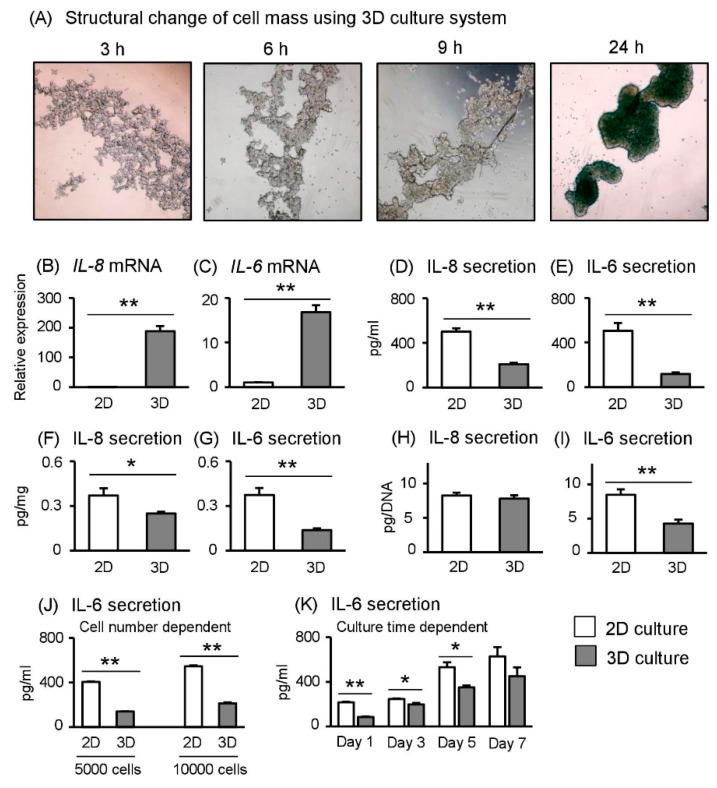
Effects of 3D culture conditions on inflammatory cytokines in Sw.71 cells. Sw.71 trophoblast cells were incubated in 2D or 3D culture plates. (**A**) Observation of structural changes of Sw.71 cells maintained in 3D (×40 magnification). (**B**,**C**) After 24 h incubation, *IL-8* and *IL-6* mRNA levels were measured using RT-qPCR (*n* = 4). (**D**,**E**) After 24 h incubation, IL-8 and IL-6 concentrations in supernatants (/mL) were determined using ELISA (*n* = 4). (**F**,**G**) After 24 h incubation, IL-8 and IL-6 concentrations in supernatants (/protein mg) were calculated (*n* = 4). (**H**,**I**) After 24 h incubation, IL-8 and IL-6 concentrations in supernatants (/DNA concentration) were calculated (*n* = 4). (**J**) After 24 h incubation, cell number-dependent IL-6 concentrations in supernatants (/mL) were determined using ELISA (*n* = 4). (**K**) Culture time-dependent IL-6 concentrations in supernatants (/mL) were determined using ELISA (*n* = 4). Data are expressed as mean ± standard error of the mean (SEM). Significant differences were detected using a *t*-test; *p* < 0.05 (*) *p* < 0.01 (**).

To aggregate human Sw.71 trophoblast cells, we used cell-repellent surface-treated cell culture plates. Time-dependent microscopy observation demonstrated that Sw.71 cells first formed a loose cell-to-cell network at 3 h that gradually became stronger cell-to-cell binding at 6 and 9 h after incubation (Figure 1A). Once aggregated completely at 24 h after incubation (Figure 1A), spheroids did not increase in size but progressively compacted until day 7 after incubation (data not shown).

### 2.2. 3D–Cultured Sw.71 Cells Possess Distinct Gene Expression Patterns Compared with 2D–Cultured Cells

Six upstream regulators were extracted from next-generation sequencing data: Tumor necrosis factor (TNF), interleukin (IL)-1β, NFκB (complex), IL-1α, IL-6, and interferon gamma. They were predicted as activated upstream regulators, although there were no inhibited upstream regulators in 3D-cultured cells. All predicted upstream regulators were inflammation-related factors.

Next, we investigated the integrated effects of cell aggregation based on the transcription levels of these genes (Supplementary Table S1: The top 30 activated genes in 3D culture cells). Several of the genes were associated with proinflammatory signaling involving *CXCL8 (IL-8)*, *CXCL1*, *CA9*, *IL-32*, *TNFAIP3*, *PTX3*, *LOX*, *MMP1*, *PLIN2*, *JUNB*, *IL-6*, *IER3*, *STMN3*, and *IRAK2*. On the other hand, several other genes were associated with anti-inflammatory signaling involving *STC1*, *SOD2*, and *GDF15*. These findings demonstrated that compared with 2D-cultured cells, gene expression related to inflammatory responses changes in 3D-cultured Sw.71 cells.

### 2.3. Spheroid Sw.71 Cells Grown under 3D Culture Conditions Reduce Inflammatory Cytokine Secretion

From above transcriptome data, we selected inflammatory cytokines IL-8 and IL-6 for downstream analysis. In agreement with our transcriptome results, *IL-8* and *IL-6* mRNA abundance significantly increased under 3D culture (1 × 10^5^ cells/well), as determined using RT-qPCR (Figure 1B,C). However, in terms of secretion concentration in the culture medium, IL-8 and IL-6 secretions were significantly decreased in spheroid Sw.71 cells in 3D-compared with 2D-cultured cells (Figure 1D,E). When calculating secretion rates for protein concentration (Figure 1F,G) and DNA concentration (Figure 1H,I), IL-6 secretion was significantly decreased in spheroid Sw.71 cells according to both calculation methods, but IL-8 secretion was not. These findings suggested that spheroid Sw.71 cells reduce inflammatory cytokine secretions, especially IL-6, whereas mRNA abundance is higher under 3D culture conditions compared with 2D culture conditions. In addition, even if the number of cells during culturing changes (Figure 1J) or the number of culturing days is extended (Figure 1K), IL-6 secretion levels clearly decreased in spheroid Sw.71 cells in the 3D culture system compared with the 2D culture system.

### 2.4. NF-κB Levels are Higher in Spheroid Sw.71 Cells

**Figure 2:**
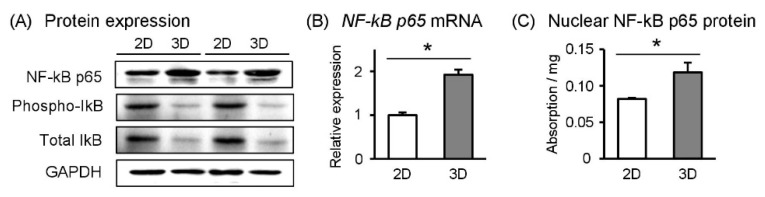
Effects of 3D culture conditions on NF-κB system in Sw.71 cells. Sw.71 trophoblast cells were incubated for 24 h in 2D or 3D culture plates. (**A**) NF-κB p65, phosphor IκB, total IκB, and GAPDH protein levels in the cell lysates were detected using Western blot. Representative data are shown. (**B**) *NF-κB p65* mRNA levels were measured using RT-qPCR (*n* = 4). (**C**) Active NF-κB p65 expression isolated from nuclei were determined using ELISA (*n* = 3). Data are expressed as mean ± SEM. Significant differences were detected using a *t*-test; *p* < 0.05 (*).

We investigated the key inflammation-associated transcription factor NF-κB [10]. To support our finding of reduced *IL-6* mRNA expression (Figure 1C), we observed that NF-κB p65 protein and mRNA expression levels were lower in Sw.71 cells maintained 2D culture condition (Figure 2A,B). Generally, inactive NF-κB complexes regulated by phospho-IκB and IκB are restricted to the cytoplasm, whereas active NF-κB complexes (p65) translocate to the nucleus [10]. We observed that nuclear NF-κB levels decreased in Sw.71 cells under 2D culture conditions (Figure 2C). In addition, phosphor-IκB and total IκB protein expressions were higher in 2D- than in 3D-cultured cells (Figure 2A). Therefore, this suggested that higher activation of NF-κB systems in spheroid Sw.71 cells under 3D culture conditions are associated with a higher expression of *IL-6* mRNA.

### 2.5. Post–Transcriptional Factor Regnase-1 More Abundant in Spheroid Sw.71 Cells

**Figure 3:**
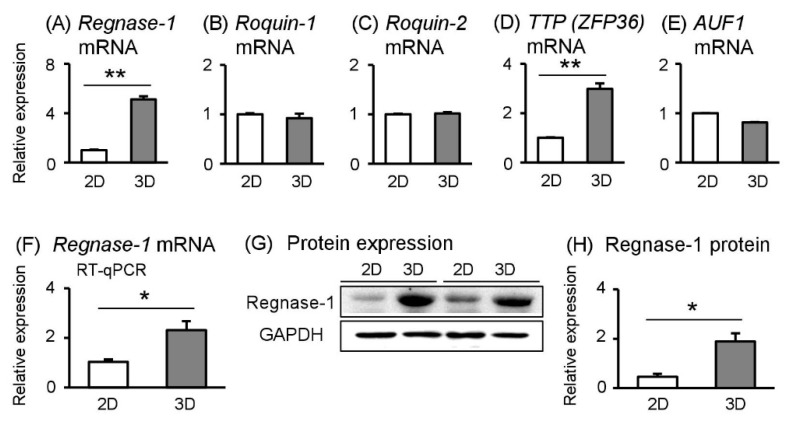
Effects of 3D culture conditions on RNase of inflammatory cytokine in Sw.71 cells. Sw.71 trophoblast cells were incubated for 24 h in 2D or 3D culture plates. (**A**–**E**) After 24 h incubation, *regnase-1*, *roquin-1*, *roquin-2*, *TTP*, and *AUR1* mRNA levels were measured using next-generation sequencing (*n* = 3). (**F**) *Regnase-1* mRNA levels were measured using RT-qPCR (*n* = 4). (**G**,**H**) Regnase-1 and GAPDH protein levels in the cell lysates were detected using Western blot. Representative data are shown. Data are expressed as mean ± SEM (*n* = 4 in each experiment). Significant differences were detected using a *t*-test; *p* < 0.05 (*) *p* < 0.01 (**).

The mRNA expression levels of various types of cytokines are controlled at both transcriptional and post-transcriptional levels [11]. The half-life of many immune-related mRNAs is short due to conserved cis-elements, including AU-rich elements (ARE) and stem-loop structures in their 3′ UTRs. Recently, the important factors that destabilize inflammation-related mRNAs have been identified, including regnase-1, roquin-1, roquin-2, tristetraprolin (TTP; also known as Zfp36), and ARE RNA binding protein 1 (AUF1) [11,12]. Therefore, we assessed mRNA expression levels of these factors by the next generation sequencing data; *regnase-1* and *TTP* mRNA expression were significantly higher in spheroid Sw.71 cells in 3D culture, whereas there was no change in mRNA expression of *roquin-1*, *roquin-2*, and *AUF1* between 2D- and 3D-cultured cells (Figure 3A–E). We focused on regnase-1 because Matsushita et al. [12] demonstrated that regnase-1 is an essential RNase for inflammatory cytokine mRNA, including IL-6. To confirm our sequencing results, we observed that *regnase-1* mRNA expression was significantly higher in 3D- than in 2D-cultured cells (Figure 3F). In addition, regnase-1 protein abundance was also significantly higher in 3D- than in 2D-cultured cells (Figure 3G,H). These findings suggested that regnase-1 negatively regulates IL-6 secretion via destabilization of *IL-6* mRNA in spheroid Sw.71 cells.

### 2.6. Spheroid Sw.71 Cells Change Cytoskeletal–Related Molecules

**Figure 4:**
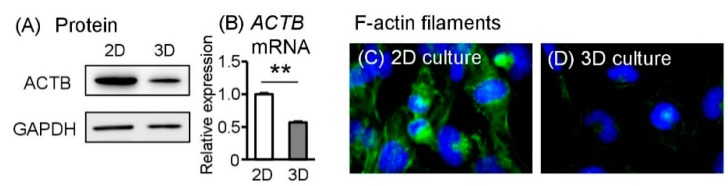
Effects of 3D culture conditions on the actin cytoskeleton in Sw.71 cells. Sw.71 trophoblast cells were incubated for 24 h under 2D or 3D culture plates. (**A**) After 24 h incubation, β-actin (ACTB) and GAPDH protein levels in the cell lysates were detected using Western blot. Representative data are shown. (**B**) *ACTB* mRNA levels were measured using RT-qPCR (*n* = 4). (**C**,**D**) Representative images of F-actin filaments staining after 2D, 3D culture (×400 magnification). Data are expressed as mean ± SEM (*n* = 4 in each experiment). Significant differences were detected using *t*-test; *p* < 0.05 (*) *p* < 0.01 (**)

Since the shape and adhesion of cells are different in 2D and 3D culture conditions, we investigated expression levels of cytoskeleton-related molecules such as β-actin (ACTB), a primary molecule of actin filament. Cultured under 2D conditions, ACTB protein was found abundance in Sw.71 cells; however, under 3D conditions, ACTB levels markedly declined (Figure 4A). Similar with our protein data, *ACTB* mRNA expression was significantly reduced in Sw.71 cells under 3D culture conditions (Figure 4B). From our pathway analysis using next-generation sequencing, we demonstrated changes in actin regulation-related molecules (Supplementary Table S2).

To observe changes in the cytoskeleton, 2D monolayers or 3D spheroids Sw.71 cells were incubated for 24 h and then trypsinized, dissociated, and re-adhered to 8-well chamber glass plates. To support the above data of protein and mRNA expressions, in 2D culture, cytoskeleton structures such as actin filaments stretched networks and were highly expressed, whereas in the 3D cultures, the cytoskeleton was loose in tension and was minimally expressed (Figure 4C,D). Thus, different from the organization of actin cytoskeleton in 3D culture remained after trypsinized and cultured as monolayer condition.

### 2.7. Actin Cytoskeleton Regulates 3D Cell Formation and IL–6 Secretion

**Figure 5:**
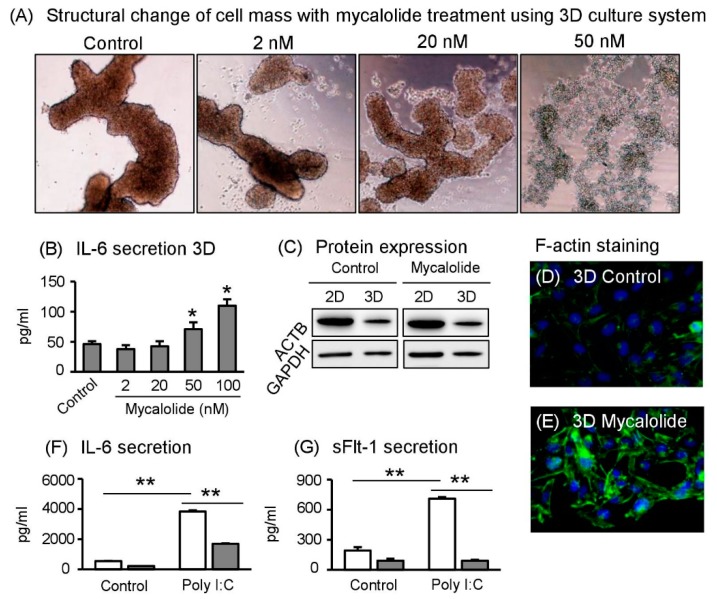
Effects of mycalolide B and poly I:C on Sw.71 cells. (**A**) Sw.71 trophoblast cells were incubated for 24 h in 3D culture plates with or without mycalolide B. Observation of structural change of Sw.71 cells under 3D culture plate (×40 magnification). (**B**) IL-6 concentrations in supernatants (/mL) treated with mycalolide B were determined using ELISA (*n* = 4). (**C**) ACTB and GAPDH protein levels in the cell lysates were detected using Western blot. Representative data are shown. (**D**,**E**) Representative images of stained F-actin filaments with or without mycalolide B treatment (x 200 magnification). (**F**,**G**) IL-6 and sFlt-1 concentrations in supernatants (/mL) from cells treated with poly I:C were determined using ELISA (*n* = 4). Data are expressed as mean ± SEM. Significant differences were detected using a *t*-test; *p* < 0.05 (*) *p* < 0.01 (**).

We hypothesized that the decrease in actin expression levels is key to form spheroid-like structures in Sw.71 cells under 3D culture conditions. To clarify the role of actin cytoskeleton, we treated spheroid Sw.71 cells with mycalolide B, an inhibitor of actin polymerization [13]. After incubation for 24 h, the mycalolide B treatment reduced cell-to-cell contact followed by 3D cell formation in a dose-dependent manner (Figure 5A). Under these conditions, IL-6 secretion significantly increased in a dose-dependent manner of mycalolide B treatment in spheroid Sw.71 cells (Figure 5B). On the other hand, the mycalolide B treatment did not affect ACTB protein expression under 2D or 3D culture conditions (Figure 5C). Interestingly, after cells re-adhered to 2D glass plates, cells treated with mycalolide B clearly expressed higher amounts of actin filament (F-actin), similar to cells maintained under 2D culture condition, compared with 3D control (Figure 5D,E).

### 2.8. Spheroid Sw.71 Cells Modulate Inflammatory Responses

Since IL-6 secretion is dependent on cell culture conditions, we finally determined whether inflammatory responses are also modified in a cell culture-dependent manner. For this aim, we treated polyinosinic:polycytidylic (Poly I:C), which is known to affect and induce inflammatory responses in Sw.71 cells [14]. Although treatment with Poly I:C significantly stimulated IL-6 secretion both in cells maintained under 2D and 3D culture conditions, stimulated levels were significantly lower in 3D- than in 2D-cultured cells (Figure 5F). Finally, we determined sFlt-1 secretion levels, because high sFlt-1levels from the placenta may contribute to the pathogenesis of preeclampsia, along with inflammation [15,16]. Interestingly, Poly I:C treated spheroid Sw.71 cells did not elicit sFlt-1 secretion, whereas Poly I:C treatment significantly stimulated sFlt-1 secretion in Sw.71 cells maintained under 2D culture conditions (Figure 5G).

## 3. Discussion

Although traditional 2D monolayer cell culture is a powerful tool to understand how cells proliferate and respond to stress, it does not recreate the in vivo 3D environment [17]. Multiple studies demonstrated that 3D organization can reveal insights into the mechanisms of complex cellular functions, including those of cancer cells. In addition, many studies investigating placental function and implantation have used the 3D culture system [2,6,18].

In the present study, at first, we compared features of 2D monolayer and 3D spheroid Sw.71 cells using next-generation sequencing. Interestingly, all predicted upstream regulators and major upregulated factors were proinflammatory signaling-related molecules in spheroid Sw.71 cells. We confirmed the upregulation of proinflammatory cytokines, including *IL-8* and *IL-6* mRNA in spheroid Sw.71 cells using RT-qPCR and compared them to similar analyses performed using 2D monolayer cells. Similarly, DelNero et al. [19] reported that proinflammatory pathways and factors, especially IL-8, increase and are essential regulators of tumor cells in 3D culture environments. However, proinflammatory cytokine secretion levels (especially IL-6) were lower in spheroid Sw.71 cells compared with cell monolayers, indicating a disconnection between cytokine gene expression and protein synthesis.

To clarify this disconnection, we focused on post-transcriptional factors that modulate inflammation by decreasing mRNA stability and protein translation efficiency [11]. Matsushita et al. [12] identified regnase-1 as an RNase critical in preventing severe autoimmune inflammatory disease in mice via destabilizing inflammation-related mRNAs. Indeed, recombinant regnase-1 directly cleaved the 3′ UTR of *IL-6*; overexpression of regnase-1 accelerated *IL-6* mRNA degradation [11,12,20]. In contrast, macrophages from regnase-1^−/−^ mice had increased IL-6 levels in response to toll-like receptor ligands, such as lipopolysaccharides [12]. In the present study, regnase-1 mRNA and protein levels were higher in 3D- than in 2D-cultured cells, suggesting that regnase-1 regulates *IL-6* mRNA stability and disassembly in the 3D culture environment, resulting in the decrease of IL-6 secretion. Further investigation is required to confirm the role of regnase-1 in these spheroid trophoblast cells.

Similar to regnase-1, roquin-1 and roquin-2 are RNA binding proteins that degrade inflammation-related mRNAs [21,22]. However, Mino et al. [21] clearly demonstrated that the post-transcriptional regulation of inflammation is spatiotemporally controlled by regnase-1 and roquin via distinct manners. They showed that regnase-1 colocalized with ribosomes and suppressed translationally active mRNAs, suggesting that regnase-1 tends to regulate the early phase of inflammation. In contrast, roquin localized with stress granules and controlled translationally inactive mRNAs, suggesting roquin plays a role in the late phase of inflammation. These findings along with our results suggested that spheroid Sw.71 cells are in an active state of translation, thereby regnase-1, but not requin (requin-1 and requin-2) may target translationally active mRNAs such as IL-6. To note, TTP, another RNA degradation regulator that also increased in spheroid Sw.71 cells. This TTP may regulate the decay of other inflammatory-related mRNAs distinct from regnase-1 targets, including CLCX1 (which was expressed higher in 3D culture than in 2D culture, Supplementary Table S1) and TNF [12].

Previous works suggested that the aggregation of cells into 3D spheroids enhances their anti-inflammatory properties [23,24]. In human mesenchymal stromal cells cultured under 3D condition, spheroid cells expressed high levels of stanniocalcin-1 (STC1), an anti-inflammatory protein [23]. To support this data, we showed that spheroid Sw.71 cells expressed higher levels of STC1 compared with monolayers maintained in 2D culture. Therefore, we suggested that anti-inflammatory properties are upregulated to adjust the mRNA and protein expression of inflammatory cytokines activated in the 3D environment.

Since the mRNA expression of inflammatory cytokines was significantly higher in the 3D culture than in 2D culture of Sw.71 cells, it was predicted that NF-κB function, a transcriptional factor of inflammatory cytokines, was also enhanced in 3D culture. As expected, NF-κB signaling, including NF-κB mRNA and protein expressions and its activity were higher in spheroid Sw.71 cells. Importantly, Widera et al. [25] reported that NF-κB controls neural stem cell aggregation. They showed increased aggregation in response to inflammatory stimuli; a pharmacological or genetic blockade of NF-κB resulted in decreased aggregation, indicating the essential role of NF-κB inducing spheroid formation. Thus, increased NF-κB signaling in spheroid Sw.71 cells may play a role in the formation of trophoblast cell aggregates.

The cytoskeleton may fluctuate because of the dynamics in a 3D culture system, such as cell assembly. Cells are intricately connected to the external environment through their cytoskeleton [26]. Cell shape, behavior, and fate are influenced by elasticity and adhesion, which affect cytoskeletal tension [27]. In this study, we showed that Sw.71 cells in 2D culture conditions had higher mRNA and protein levels of ACTB and exhibited multiple-directed F-actin stress bundles. In contrast, the actin cytoskeleton was released in spheroid Sw.71 cells and very thin actin filaments were observed. Similar with our present data, Zhou et al. [26] reported lower expression of actin cytoskeleton tension in the 3D culture of human mesenchymal stem cells compared with 2D-cultured cells, suggesting that 3D culture released cytoskeletal tension that is present in conventional 2D culture systems, and induced morphological and mechanical changes.

To clarify the role of cytoskeletal tension in cell aggregation and inflammatory cytokine secretion, we treated spheroid Sw.71 cells with mycalolide B (an inhibitor of actin polymerization). With actin polymerization and depolymerization inhibited in 3D cultures, spheroid formation of Sw.71 cells was suppressed and IL-6 secretion increased, simulating profiles observed in 2D cultured cells. Interestingly, mycalolide B treatment did not affect actin protein expression, but actin filament was observed in similar manner seen in 2D-cultured cells. Recently, Zhou et al. [26] reported mesenchymal stem cells cultured under 3D conditions showed a relaxation of the cytoskeleton tension, reduced actin filaments, and increased expression of NANOG, along with reduced levels of H3K9 methyltransferase. In addition, they showed that pharmacological inhibition of actin polymerization with cytochalasin D elevated NANOG expression via H3K9 demethylation, suggesting epigenetic regulation via actin filament and 3D-cell formation. These collective findings suggested that actin filament in spheroid Sw.71 cells may control inflammation via epigenetics. Further research is needed to clarify the role of the cytoskeleton in spheroid trophoblast cells maintained in 3D cultures.

Finally, we compared the differential responses to immune-stimulation between 2D- and 3D-cultured cells. Treatment with Poly I:C clearly induced distinct responses, including IL-6 and sFlt1 secretion, from Sw.71 cells. Indeed, the 2D monolayer cells had elevated reactive oxygen species (ROS) and induced chronic inflammatory response due to increased cellular toxicity from absorbed nanomaterials, whereas 3D spheroids had a reduced response to poly I:C treatment [14]. Moreover, many reports demonstrated that different cellular responses between 2D and 3D culture conditions, including inflammation, NF-κB translocation, inflammatory response-related receptors, ROS production, and cellular toxicity [19,23,28,29,30,31].

In summary, our results demonstrated that dimensionality plays an essential role in governing spatiotemporal cellular outcomes, including inflammatory cytokine production and regnase-1 mediated regulation. Three dimensional cell culturing is gaining popularity, especially in placental physiology and implantation because it sheds light on physiological complexities. To further enhance physiological relevance, trophoblast cells could be cocultured with immune cells or endothelial cells to create the 3D microenvironment of human placenta, which would provide a powerful tool for studying cellular cross-talk present in this milieu.

## 4. Materials and Methods

### 4.1. Human Trophoblast Cell Culture

Human first-trimester trophoblast cells (Sw.71, trophoblast cell line) produced various types of cytokines, such as IL-6 and IL-8 [9,32] were kindly provided by Professor Gil Mor [8]. Cells were cultured in the DMEM/F-12 (Life Technologies Corporation, Carlsbad, CA, Canada) supplemented with antibiotics (Amphotericin B and Gentamicin; Sigma-Aldrich, St Louis, MO, USA), sodium pyruvate (Wako Pure Chemical Industries, Ltd., Osaka, Japan), non-essential amino acids (Wako Pure Chemical Industries, Osaka, Japan), and 5% heat-inactivated fetal calf serum (FCS; ICN, Costa Mesa, CA, Canada).

Cells were incubated at concentrations of 0.fig cells/well for 1–7 days in 24-well culture plates in 5% FCS condition medium. For 2D cultures, cell suspensions were plated on nunclon delta treated-cell culture plates (Thermo Fisher Scientific, Tokyo, Japan). For 3D cultures, cell suspensions were plated on cell-repellent surface treated-cell culture plates (Greiner Bio-One, GmbHm, Frickenhausen, Germany). Then, supernatants were collected to detect the secretion of cytokines by ELISA and cell lysates were collected for mRNA and protein expression analyses.

To investigate the role of the actin cytoskeleton, a Sw.71 cell suspension was placed on 3D culture plates with or without mycalolide B (Wako Pure Chemical Industries) [13] for 24 h. In addition, to investigate the differences in inflammatory responses between 2D and 3D culture systems, cell suspensions were placed on 2D or 3D culture plates with or without polyinosinic-polycytidylic acid (Poly I:C, a agonist of toll-like receptor 3; 100 µg/mL; InvivoGen, San Diego, CA, USA) for 24 h.

### 4.2. Transcriptome Analysis of Human Trophoblast Cells

To clarify differences in trophoblast cell characteristics between 2D and 3D culture systems, we performed transcriptome analyses using next-generation sequencing (Illumina; San Diego, CA, USA) as previously described [33]. Simply, cells were plated at a concentration of 1 × 10^5^ cells/well in 2D or 3D culture plates for 24 h. After incubation, the cells were washed twice with PBS and total RNA was extracted using an RNAqueous RNA Isolation Kit (Thermo Fisher Scientific) according to the manufacturer’s instructions. After assessing RNA quality using a 2100 Bioanalyzer (Agilent Technologies, Palo Alto, CA, USA), libraries were prepared with a TruSeq RNA Sample Preparation Kit v2 (Illumina). Using these libraries, clusters were generated with an Illumina cBot and two lanes for the two groups were sequenced as 100-base reads (single end) by an Illumina HiSeq 2500 Sequencing System. Bcl2fastq2 ver. 2.17 (Illumina) was used for image analysis, base calling, and quality filtering according to the manufacturer’s instructions. Derived sequence data were aligned with the human genome sequence (GRCh38) to count the sequence reads using CLC Genomics Workbench (Qiagen; Redwood City, CA, USA). Significantly differentially expressed genes were analyzed using upstream regulators in Ingenuity Pathways Analysis (IPA) program (Qiagen). IPA was used to identify biofunctional signaling networks associated with differential changes in gene expression between 2D and 3D culture. Analysis was carried out with *n* = 3 in each group. Kal’s *z*-test was used to analyze gene set enrichment in the functional categories. The data used for this analysis comprised a gene list of 24,094. The data were further filtered with IPA at a fold-change threshold of 2.0 and false-discovery rate adjusted *p*-value < 0.05.

Using IPA analysis software, 257 upstream regulators were predicted from the differentially expressed genes in cells between 2D and 3D culture systems (data not shown). We determined the activation state of an upstream regulator based on gene regulation within the comparison gene sets. The predicted upstream scores were determined, and for upstream factors with scores greater than 6 or less than -6 (6 fold changes), a *p-*value < 0.0001 was selected.

### 4.3. Real–Time RT-PCR

After 24 h incubation, total RNA was prepared using ISOGEN (Nippon Gene Company, Limited, Toyama, Japan) according to the manufacturer’s instructions. Real-time quantitative reverse transcription-polymerase chain reaction (RT-qPCR) was performed using the CFX Connect^TM^ Real Time PCR (Bio-Rad, Hercules, CA, USA) to detect mRNA expressions of IL-6, IL-8, nuclear factor-kappa B (NF-κB p65), regnase-1 or Ribosomal Protein Lateral Stalk Subunit P0 (RPLP0) as an endogenous control. The following antisense and sense primers were used: *IL-6* (5′- AAA TTC GGT ACA TCC TCG ACG G -3′ and 5′- GGA AGG TTC AGG TTG TTT TCT GC -3′), *IL-8* (5′- CTT GGC AGC CTT CCT GAT TTC -3′ and 5′- GGT GGA AAG GTT TGG AGT ATG TCT -3′), *NF-κB p65* (5′- ATC CCA TCT TTG ACA ATC GTG C -3′ and 5′- CTG GTC CCG TGA AAT ACA CCT C -3′), regnase-1 (5′- GGA AGC AGC CGT GTC CCT ATG -3′ and 5′- TCC AGG CTG CAC TGC TCA CTC -3′) and *HPRT1* (5′- GAG ATG GGA GGC CAT CAC ATT GTA GCC CTC -3′ and 5′- CTC CAC CAA TTA CTT TTA TGT CCC CTG TTG ACT GGT C -3′). RT-qPCR was performed in duplicate with a final reaction volume of 20 µL containing 10 µL SYBR Green (Thunderbird SYBR qPCR Mix; Toyobo Co., Ltd., Osaka, Japan), 7.8 µL distilled water, 0.1 µL 100 µM forward and reverse primers, and 2 µL of complementary DNA template. The amplification program consisted of a 5 min denaturation at 95 °C followed by 40 cycles of amplification (95 °C for 15 s, 60 °C for 30 s, and 72 °C for 20 s). Expression levels of each target gene were normalized to corresponding *HPRT1* threshold cycle (CT) values using the ΔΔ CT comparative method [34]. The specific melting point of the amplified product carried out as verification of the product identify. After real-time RT-PCR analysis, the PCR products were subjected to electrophoresis, and the target band was observed in the predicted size. 

### 4.4. Determination of Cytokines

After incubation in each culture system, supernatant was collected and stored at −20 °C before use. Levels of IL-6, IL-8, or soluble fms-like tyrosine kinase-1 (sFlt1 as a diagnostic marker of preeclampsia) were determined using a human ELISA kit (R&D Systems, Minneapolis, MN, USA) according to the manufacturer’s instructions. The results represent at least four independent experiments.

### 4.5. NF-κB p65 Activation Assay

After incubation, the nuclei and cytosol of cultured Sw.71 were separated using a nuclear extraction kit (Abcam, Cambridge, UK) according to the manufacturer’s instructions. Generally, inactive NF-κB complexes are existed in the cytoplasm, whereas active NF-κB complexes (p65) translocate to the nucleus [10]. Therefore, the expression of NF-κB p65 levels in isolated nuclei and cytosol were analyzed using the NF-κB p65 transcription factor assay kit (Abcam) according to the manufacturer’s instructions [35]. Absorbance at a wavelength of 450 nm was measured to assay for protein expression levels and the protein concentration was then adjusted. The results represent at three independent experiments.

### 4.6. Western Blot Analysis

Lysates from the cell culture were prepared using RIPA buffer (Wako Pure Chemical Industries). Cells were washed with cold PBS and incubated with RIPA buffer for 15 min on ice. Cell lysates were subsequently transferred into 1.5 mL tubes and centrifuged at 12,000× *g* for 20 min at 4 °C. Supernatants were transferred to a fresh tube and stored at −80 °C before analysis. A total of 10 µg protein was loaded per lane and separated by 12% sodium dodecyl sulfate-polyacrylamide gel electrophoresis (SDS-PAGE). The expression of NF-κB p65, phosphor-IκB, IκB, Regnase-1, GAPDH, and β-actin (ACTB) was analyzed by Western blot. After transfer onto polyvinylidene fluoride membranes, nonspecific antibody binding was blocked for 1 h at room temperature using Immunoblock (DS Pharma Biomedical Co., Ltd., Osaka, Japan). Then, membranes were incubated for 24 h at 4 °C with anti-NF-κB p65 antibody (1:1000, Cell Signaling Technology, Inc., Danvers, MA, USA), anti- phosphor-IκB antibody (1:200, R&D systems Inc., Minneapolis, MN, USA), anti-IκB antibody (1:5000, R&D systems), anti-Regnase-1 antibody (1:500, R&D systems), anti-GAPDH antibody (1:2500, Abcam), and anti-ACTB antibody (1:10,000, Cell Signaling Technology), followed by an incubation for 1 h with secondary antibody conjugated horseradish peroxidase (HRP; 1:1000, GE Healthcare, UK Ltd., Buckinghamshire, UK). Immunoreactive bands were visualized by Western BLoT Quant HRP Substrate (GE Healthcare) using ImageQuant LAS 4000 (GE Healthcare). The results represent at least three independent experiments. Quantitative analysis of bands was performed using Image J (National Institutes of Health, Bethesda, MD, USA).

### 4.7. Immunocytochemistry

After incubation in each culture system, Sw.71 cells were re-seeded in 8-well chamber glass dishes (Merck Millipore, Temecula, CA, Canada) and further cultured for 24 h. After blocking with 5% BSA in PBS for 1 h, the cells were incubated with Alexa 488 fluorescent phalloidin (Cytoskeleton, Inc., Denver, CO, USA) for 90 min. The cells were covered with VECTASHIELD with DAPI (Vector Laboratories, Inc., Burlingame, CA, USA), and staining sections were analyzed using a confocal microscope (Leica Mycrosystems, Inc., Tokyo, Japan).

### 4.8. Statistical Analysis

Data are expressed as mean ± standard error of the mean (SEM). Differences between 2D and 3D culture systems were identified using unpaired *t-*tests. A *p*-value of <0.05 was considered to be statistically significant.

## Supplementary Materials

The following are available online at http://www.mdpi.com/1422-0067/19/8/2322/s1, Table S1: Upregulated genes in 3D culture, Table S2: Actin regulation-related molecules.
